# Perception and awareness of unintentional childhood injuries among primary caregivers of children in Vellore, South India: a community-based cross-sectional study using photo-elicitation method

**DOI:** 10.1186/s40621-020-00289-4

**Published:** 2020-12-14

**Authors:** Leeberk Raja Inbaraj, Kulandaipalayam Natarajan Sindhu, Lalmalsawmi Ralte, Basir Ahmed, Chandni Chandramouli, Elza Rebecca Kharsyntiew, Evelina Jane, Joshaphine Victoria Paripooranam, Nikhil Muduli, Padebettu Devendra Akhilesh, Prakash Joseph, Renata Nappoly, Tamma Anusha Reddy, Shantidani Minz

**Affiliations:** 1 Division of Community Health, Bangalore Baptist Hospital, Bangalore, Karnataka 560024 India; 2 Department of Community Health, Christian Medical College, Vellore, Tamil Nadu India; 3 The Wellcome Trust Research Laboratory, Division of Gastrointestinal Sciences, Christian Medical College, Vellore, Tamil Nadu India; 4 Rural Unit for Health and Social Affairs, Christian Medical College, Vellore, Tamil Nadu India

**Keywords:** Unintentional injury, Children, Primary caregivers, Rural

## Abstract

**Objective:**

We studied the primary caregivers’ perception, and further, their awareness of unintentional childhood injuries in south India.

**Methods:**

A cross-sectional study was conducted in the rural block of Kaniyambadi, Vellore, among 300 primary caregivers of children aged between 0 and 14 years. A semi-structured interview was conducted with the primary caregivers using a photo-elicitation method, with a visual depiction of ten injury risky scenarios for a child. Scoring was done to assess the perception of environmental hazards in these scenarios, and further, knowledge on the prevention of these injuries. An independent ‘t’ test was done to elicit differences in mean scores and a multivariate regression analysis was applied to ascertain factors independently associated with the scores.

**Results:**

Primary caregivers had adequate perception regarding risks posed to children in scenarios such as climbing trees (96.2%), playing near construction sites (96%), firecrackers (96.4%) and crossing unmanned roads with no traffic signals (94%). Knowledge of prevention was poor however, in the following scenarios: a woman riding a bicycle without safety features, with child pillion sitting behind bare foot and legs hanging by one side (72.6%); a child playing near a construction site (85.9%); and a child playing with plastic bags (88.3%). Overall, educational status of the primary caregiver and socioeconomic status were associated with poorer perception of risks and knowledge about unintentional childhood injuries and their prevention.

**Conclusions:**

Pragmatic community-based childhood interventions incorporated into existing programs, with a special focus on road traffic injuries, burns and suffocation need to be implemented in high-risk settings of rural populations in South India.

## Introduction

The Global Burden of Disease (GBD) study estimated that unintentional injuries (UI) contributed to 627,741 (18%) of the 3.5 million deaths among the 1–19 year-olds in 2010 (Kassebaum et al. 2017). The World Health Organization (WHO) further estimated that the injury-specific mortality in the under-five age bracket was 73 per 100,000 population (WHO 2015). Road traffic injuries (RTIs), drowning, burns, falls and poisoning were listed as the five leading causes of injury-related deaths among children (Linnan et al. 2007). The Million Death Study (MDS) revealed that among children under 4 years and between 5 and 14 years in India, injuries contributed to 3.2 and 16% of deaths, respectively (Jagnoor et al. 2011).

Both the urban as well as rural settings have a high burden of unintentional childhood injuries as shown by studies from central and south India (Sharma et al. 2018; Mathur et al. 2018). However, the types of injuries encountered in both the settings were quite different. In rural areas, there was a higher incidence of agricultural injuries and poisoning, whereas in urban areas it mostly consisted of falls, burns and road traffic injuries (Sharma et al. 2018; Mathur et al. 2018).

While data on the burden and epidemiology of unintentional childhood injuries in low- and -income countries (LMICs) is just beginning to emerge, data on injury perception and knowledge among the primary caregivers is very limited in these settings (Mathur et al. 2018). It was found that lack of parental supervision was associated with deaths due to drowning, pedestrian injuries and falls in children aged 0–14 years (Morrongiello 2005). The knowledge and perception of these injuries is vital in the prevention of the same, while taking into account the parenting experiences and beliefs that influence child supervision practices (Petrass et al. 2009). Parental perceptions determine situations being perceived as risky or risk-free and are thereby critical in injury prevention. Evidence suggests that a lack of parental knowledge about unintentional childhood injuries is an important factor related to failure in adopting safe practices (McKenzie et al. 2019). The “Health Belief Model” states that preventive behaviours are a result of people’s beliefs about their susceptibility to the health problem, the severity of the health problem, and the cost-versus-benefit of adopting the safe behaviour (Janz and Becker 1984). Peterson et al. (1990) used this model to predict how parents’ attitudes can influence injury prevention teaching and environmental modifications.

A majority of the primary caregivers in low, and surprisingly, even in high- income settings a significant proportion of them believe that these injuries generally cannot be prevented (Siu et al. 2019; Mulligan-Smith et al. 1998). A study from Bangladesh showed that it was believed that drowning was a “natural and inevitable” incident and not a preventable unintentional childhood injury (Rahman et al. 2008). A qualitative study using focus group discussions in two low-income settings in South Africa showed that parents attributed these injuries more to the environment and lacked insight into individual prevention strategies that could be employed such as parental supervision (Munro et al. 2006). Further, merely emphasizing parental supervision in rural settings may seem more theoretical given that the primary caregivers are busy with their household chores or work (Siu et al. 2019).

Nevertheless, evidence does show that home safety education plays a significant role in the prevention of these injuries on par with the provision of home safety interventions. However, this evidence from a systematic review was done within the purview of industrialized settings (Kendrick et al. 2013). Environmental modification with home safety intervention is generally non-feasible from a technical perspective, and challenging in the low-income settings. It was concluded that the “health belief” behavioural change approach can be used in targeted educational interventions to bring about desired behavioural outcomes (Gielen and Sleet 2003). While another behavioural theory, “Applied Behavioural Analysis,” which sought to understand and modify behaviour by addressing antecedents, behaviour and consequences (ABCs), produced consistent positive results in injury prevention (Gielen and Sleet 2003). We suggest that a pragmatic and feasible approach would, no doubt, be the education of the primary caregivers in these settings, without denying the fact that injury prevention is a multi-pronged approach. However, this must first begin with a deep understanding of the caregiver’s perception and awareness of unintentional childhood injuries.

Since there is no formal injury surveillance system in India, there is a definite need to study childhood injuries and their prevention, beginning with exploring the perceptions and knowledge among the primary caregivers of children. In general, conventional survey methods have been used to explore knowledge and perception of unintentional childhood injuries without specifically taking into consideration the type of the setting and its cultural norms (Meo 2010; Inbaraj et al. 2017; Hogan et al. 2018; Pant et al. 2014). While our study’s aims were the same, we instead used a photo-elicitation method that is culture- and context-specific, aiming to elicit perceptions in a rural community where low literacy could potentially alter responses in questionnaire-based oral interviews.

## Methods

### Study setting

This study was conducted in Kaniyambadi Block, a rural block in Vellore district of Tamil Nadu, South India. The Department of Community Health, Christian Medical College, Vellore, has been working with the Kaniyambadi community over the last 60 years, predominantly in the areas of maternal and child health through the Community Health and Development (CHAD) program, and its secondary care hospital care service– the CHAD hospital, a secondary care hospital. Kaniyambadi block houses a population of 116,241, is predominantly agrarian, with a male and female literacy of 80 and 67%, respectively (Kaniyambadi, Vellore, Tamil Nadu 2019). Each sub-block in Kaniyambadi is catered to by a team comprised of a part-time community health worker (PTCHW), a health aide (HA) and a public health nurse (PHN). The health surveillance data of the block is maintained in the electronic database of the CHAD server as the CHAD Health Information System (HIS).

### Study design

This cross-sectional study was conducted in the Kaniyambadi block among primary caregivers of children aged between 0 and 14 years. A European study found that ~ 75% of mothers had an adequate perception of unintentional childhood injuries (Vincenten et al. 2005). Incorporating the female literacy rate in the rural Indian setting, we assumed that 40% of the primary caregivers would have adequate perception of injuries. Hence, the sample size was calculated to 300, with a relative precision of 20%, incorporating a design effect of 2. We chose six villages from Kaniyambadi block known to have the highest mortality due to unintentional childhood injuries in the last 5 years. Information on households with children aged between 0 and 14 years with precise location and way-points were extracted from the CHAD HIS. We planned to interview 50 primary caregivers from each of the six villages. By systematic random sampling, households were visited with address and eligibility being confirmed by the health aide and interviewer. Houses found to be locked at the time of the interview were visited again, and if locked for the second time, the particular house was excluded from the sampling list. Written informed consent in the local language Tamil (a Dravidian language spoken in the South Indian state of Tamil Nadu) was obtained from the primary caregiver before the interview.

### Study tool and data collection

**Fig. 1:**
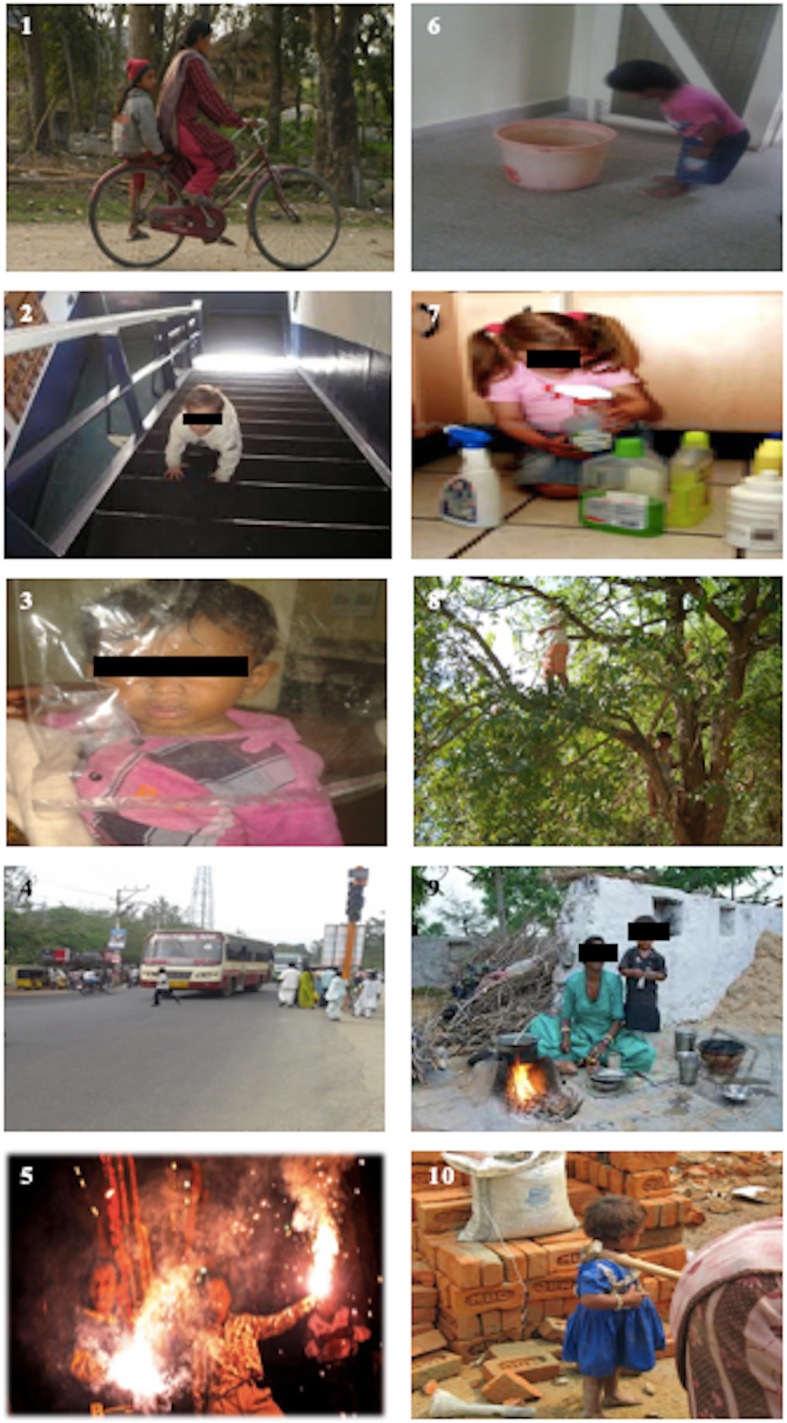
The ten scenarios depicting unintentional childhood injuries used in the photo-elicitation method while interviewing the parent/primary caregiver

A semi-structured interview using a photo-elicitation method, a visual narrative method, was used to elicit the awareness of unintentional childhood injuries in the community. The study instrument thereby comprised of two parts: the first contained questions on socio-demographic profile of the primary caregivers: age, gender, education, occupation and socioeconomic status (SES); and the second contained photographs depicting ten scenarios of unintentional injuries as shown in Fig. 1. SES was assessed using the modified Kuppuswamy scale (Bairwa et al. 2013).

The modified Kuppuswamy scale is a measure of socio-economic status of an individual based on three variables: education, occupation of the head of the family, and per capita income of the family per month (Bairwa et al. 2013). The ten scenarios used in the photo-elicitation method included the following: a woman riding a bicycle without safety features (skirt guard and reflectors) with a child pillion sitting behind (riding double / riding two-up) barefoot and legs hanging by one side with no helmet; a toddler climbing a staircase without supervision; an infant playing with a plastic bag; a boy crossing the road in a non-zebra crossing; small children playing with firecrackers; an unsupervised toddler near a bucket of water; an infant playing with household chemicals at home; small children climbing trees; a mother cooking food using a firewood stove placed at the ground level; and an unsupervised child playing at a construction site. The primary caregivers were asked the following questions when each photo of each of the above scenarios was shown to them:

- To assess caregiver’s perception of the hazard posed by the scenario, the first question was: “Can this scenario lead to injury to the child?” (yes/no response). If the answer was ‘Yes,’ the next question was an open-ended question to assess the knowledge on the type of injuries: “What are the types of injuries that can occur?”
- Similarly, a third question was used to assess caregiver’s perception of prevention: “Can the injuries mentioned by the primary caregiver be prevented?” (yes/no response) If the response was ‘Yes,’ the final question to assess the knowledge on preventive measures as an open-ended question: “What are the measures to prevent these injuries?”

If the response to the first question was ‘No,’ the participants were not asked any further questions for that particular scenario.

### Data quality assurance

The questionnaire was translated into Tamil by two different people well-versed with the local dialect. A back-translation was done to English to ensure accuracy. The questionnaire including the photo-elicitation technique was first pilot-tested among 10 primary care givers in a village (the data of which was not included in the study), following which necessary modifications were made on the few colloquial terms that were familiar to the rural population. A 10% sample of the interview process was supervised by the principal- and co-investigators to eliminate interviewer bias. The interviews with the primary caregivers were conducted by medical students. They were trained to conduct interviews and administer the photo-elicitation method in the class room for an hour and in the field for 4 h through lectures, role plays and simulation. They were divided into five groups and the first five interviews of each group were supervised.

### Statistical analysis

Data entry was done using Epi-info version 7.0 developed by Centre for Disease Control. Analysis was done using Statistical Package for Social Services (SPSS Inc. Released 2008. SPSS Statistics for Windows, Version 17.0. Chicago: SPSS Inc.). Each variable was summarized by frequency count. The frequency distribution of responses to variables such as perception of hazards, knowledge of injuries, and perception of prevention and knowledge of prevention of injuries were computed as percentages. Each correct response was given a score of 1. The scoring for the responses on the open-ended questions were decided based on a consensus during three in-person meetings by a team of senior investigators. Similar methodology was followed to categorize responses from open-ended narrations. The maximum score that could be obtained for each variable was 10. Socio-demographic characteristics such as age, occupation, education, relationship to the child, type of family, SES, total number of children in the household, history of injury in the past were considered as exposure variables. An independent ‘t’ test was performed to elicit differences in the mean score. The open-ended responses were analyzed, and most common responses for knowledge of injuries and its prevention were reported.

A multivariable regression analysis was performed to ascertain the exposure factors independently associated with perception, knowledge and awareness of unintentional childhood injuries. Variables with a *p*-value ≤0.20 in the bivariate analysis were used in the final model. Statistical significance was declared at a 95% CI (confidence interval) and a *p*-value < 0.05. Collinearity between the variables was assessed by considering the variance inflation factor (VIF). VIF > 10 was assumed to be suggestive of the presence of multicollinearity.

### Ethical consideration

This study was approved by the Institutional Review Board (IRB) of Christian Medical College, Vellore (IRB min No 12622).

## Results

Overall, 302 primary caregivers of children aged between 0 to 14 years were interviewed from the six villages of Kaniyambadi block in the month of March 2013.

### Socio-demographic characteristics

The baseline characteristics of the primary caregivers interviewed are described in Table 1. Mothers (93%) were the predominant primary caregivers, followed by grandmothers (5.2%). A majority of the primary caregivers (62.9%) were aged between 18 to 30 years [mean (SD) 31 (9.2) years]. More than two-thirds (69.6%) of the primary caregivers had completed high school, with 10% of them having completed their higher education. The majority were housewives (79.8%). Nearly half of them belonged to the lower (lower and upper-lower) class (48.4%) and were from nuclear families (51.7%). A nuclear family is defined as a unit composed of a couple with and their unmarried children, a joint family refers to a couple along with their parents and unmarried children, and an extended family consists of a couple and their adult sons, their wives and children and younger children of the paternal couple.

**Table 1:** Socio-demographic characteristics of the primary caregivers interviewed from the six villages of Kaniyambadi block (*n* = 302

Variable	Category	n	%
Age (completed years)	18–30	190	62.9
	31–40	79	26.1
	41–50	19	6.2
	51–60	8	2.6
	> 60	6	1.9
Relationship to the child	Mother	281	93
	Father	1	0.3
	Aunt	3	0.9
	Grandmother	16	5.2
	Grandfather	2	0.6
Total number of children in the household	1	91	30.1
	2	165	54.6
	3	40	40
	4	6	6
Education	No schooling	13	4.3
	Primary	42	13.9
	Middle	57	18.9
	High school	98	32.5
	Post high school	59	19.5
	Degree/Diploma	24	7.9
	Professional degree	9	3.0
Occupation	House wife	241	79.8
	Unskilled worker	33	10.9
	Others	28	9.2
Type of family	Nuclear	156	51.7
	Joint	107	35.4
	Extended	39	12.9
Socioeconomic status (SES)^a^	Lower	2	0.7
	Upper lower	144	47.7
	Lower middle	112	37.1
	Upper middle	37	12.3
	Upper	7	2.3

^a^ Modified Kuppuswamy scale

### Perception of hazard and prevention of injuries

Overall, a vast majority (> 80%) of the primary caregivers had an adequate perception regarding the 8 of the 10 hazards depicted in scenarios of the photo-elicitation method. This includes scenarios such as the unsupervised climbing of trees by small children (97.2%), playing near the construction site (96%), using firecrackers (96.4%), crossing roads at non-zebra crossings (94%), and infants playing with household chemicals (91.2%) (Table 2).

**Table 2:** Perception and knowledge of unintentional injuries and their prevention elicited using the photo-elicitation method among the primary caregivers (*N* = 302)

Type of unintentional childhood injury	Adequate perception of the hazard n (%)	Adequate knowledge of type of injury n (%)	Adequate perception of prevention of injury n (%)	Adequate knowledge of prevention of injury n (%)
Woman riding bicycle without safety features, with child pillion sitting behind bare foot and legs hanging by one side without helmets	198/302 (65.6)	188/198 (94.9)	168/198 (84.8)	122/168 (72.6)
		188/302 (62.2)	168/302 (55.6)	122/302 (40.3)
Toddler climbing a staircase without supervision	272/302 (90.1)	270/272 (99.2)	252/272 (92.6)	239/252 (94.8)
		270/302 (89.4)	252/302 (83.4)	239/302 (79.1)
Infant playing with a plastic bag	228/302 (75.5)	206/228 (90.3)	215/228 (94.2)	190/215 (88.3)
		206/302 (68.2)	215/302 (71.2)	190/302 (62.9)
Boy crossing the road in a non-zebra crossing	285/302 (94.4)	285/285 (100)	263/285 (92.2)	245/263 (93.1)
		285/302 (94.4)	263/302 (87.1)	245/302 (81.1)
Small children playing with firecrackers	291/302 (96.4)	290/291 (99.6)	260/291 (89.3)	243/260 (93.4)
		290/302 (96)	260/302 (86.1)	243/302 (80.5)
Unsupervised toddler near a bucket of water	265/302 (87.7)	261/265 (98.4)	256/265 (94.3)	238/256 (92.9)
		261/302 (86.4)	256/302 (84.8)	238/302 (78.8)
Infant playing with household chemicals	275/302 (91.2)	268/275 (97.4)	265/275 (96.3)	246/265 (92.8)
		268/302 (88.7)	265/302 (87.7)	246/302 (81.5)
Small children climbing trees	294/302 (97.2)	294/294 (100)	272/294 (92.5)	257/272 (94.4)
		294/302 (97.2)	272/302 (90.1)	257/302 (85.1)
Mother cooking using firewood stove placed at ground level	261/302 (86.4)	259/261 (99.2)	247/261 (94.6)	228/247 (92.3)
		259/302 (85.7)	247 /302 (81.7)	228/302 (75.4)
Unsupervised child playing at a construction site	290/302 (96)	286/290 (98.6)	278/290 (95.8)	239/278 (85.9)
		286/302 (94.7)	278/302 (92)	239/302 (79.1)

However, they had a lower proportion of primary care givers perceived perception situations such as pillion riding on two-wheeler vehicles without helmets and skirt guards (65.6%), and playing with plastic bags (75.5%) were hazardous. Those who had an adequate perception regarding the aforementioned injuries also had an adequate perception of injury prevention of playing with household chemicals (96.3%) and unsupervised play near construction sites (95.8%). A significant proportion of the primary caregivers felt that injuries caused by scenarios such as pillion bicycle riding (44.4%), infants playing with plastic bags (28.8%) and firecrackers (33.9%) cannot be prevented.

The various responses obtained on the type of unintentional childhood injury/ies that could be encountered in a given scenario and measures that could be taken to prevent them are summarized in Table 3.

**Table 3:** Most common responses elicited on the type of unintentional childhood injury/ies that could possibly encountered in the given scenario and responses and measures that could potentially be taken to prevent them

Scenario	Possibility on types injuries	Preventive measures
Woman riding bicycle without safety features, with child pillion sitting behind barefoot and legs hanging by one side without helmets	• Fall from bicycle • Injury to the neck following a fall • Spokes causing injury to legs • Clothes can get stuck between the wheels	• Using skirt-guard • Avoid taking children as pillion riders • Appropriate support for the kid • The child should put legs on both sides of bicycle • Use a child seat
Toddler climbing a staircase without supervision	• Fall from staircase causing injury, especially head injury	• Supervision is essential
Infant playing with a plastic bag	• Suffocation • Can obstruct vision causing fall	• Keep plastic bags out of reach of children • Supervision • To avoid using plastic bags • To dispose plastic bags appropriately
Boy crossing the road in a non-zebra crossing	• Can be hit by a vehicle	• Accompany the child while crossing the road especially in non-zebra crossings
Small children playing with firecrackers	• Can cause burns and injury to eyes • Explosion • Poisoning due to gun powder	• Adult supervision • Using personal protective equipments • Small children should not be allowed to play with crackers • Segregate the crackers according to the age
Unsupervised toddler near a bucket of water	• Child can drown • Spillage of water, child can slip and fall	• Keep buckets/containers with water covered • Keep buckets empty
Infant playing with household chemicals	• Accidental poisoning • Inhalation of chemicals • Skin/burn injury	• Adult supervision
Small children climbing trees	• Fall from tree	• Adult supervision
Mother cooking food using firewood stove placed at the ground level	• Burn injury	• Supervision • Keep the child away from the kitchen
Unsupervised child playing at a construction site	• Brick and construction material falling on the child causing serious injury • Inhalation of cement/dust	• Supervision

### Factors associated with the perception of hazards and prevention of injuries

Mothers as primary caregivers [mean (SD) 8.5 (1.6)], having higher education [mean (SD) 9 (1.5)] and belonging to higher socioeconomic status [mean (SD) 9 (1.5)] scored better on the perception of hazards compared to primary caregivers other than mothers [mean (SD) 8 (2.4)], having lower education [mean (SD) 8.3 (1.9)] and belonging to low socioeconomic status [mean (SD) 8.3 (1.8)], with these differences being statistically significant (Table 4). However, perception of prevention of injuries was independently associated with the education of the primary caregiver (*p* = 0.001) and their socioeconomic status (*p* = 0.02) in multivariate analysis (Table 5).

**Table 4:** Factors associated with poor perception of hazard, knowledge of type of injury, and perception and knowledge on the prevention of unintentional childhood injuries

Factors	Category	n	Perception of hazards			Knowledge of type of injury			Perception of prevention			Knowledge of prevention		
			Mean	SD	***p***-value	Mean	SD	***p***-value	Mean	SD	***p***-value	Mean	SD	***p***-value
Age of primary caregiver	<=30	190	8.8	1.7	0.7	8.6	1.7	0.7	8.2	2.2	0.8	7.4	2.7	0.8
	> 30	112	9.7	1.7		8.5	1.7		8.1	2.3		7.4	2.7	
Relationship to child	Mothers	281	8.5	1.6	0.03*	8.7	1.6	0.02*	8.2	2.2	0.1	7.4	2.7	0.4
	Others	21	8.0	2.4		7.8	2.2		7.4	2.8		7.0	2.8	
Education of primary caregiver	Lower	112	8.3	1.9	< 0.001*	8.0	1.9	< 0.001*	7.4	2.5	< 0.001*	6.5	2.8	< 0.001*
	Higher	190	9.0	1.5		8.9	1.5		8.6	1.9		7.9	2.4	
Occupation of primary caregiver	Housewife	241	8.7	1.7	0.8	8.6	1.8	0.8	8.2	2.3	0.7	7.5	2.7	0.2
	Mothers	61	8.8	1.4		8.6	1.5		8.1	2.2		7.0	2.7	
Type of family	Nuclear	156	8.7	1.7	0.5	8.6	1.7	0.9	8.1	2.3	0.4	7.5	2.7	0.4
	Non nuclear	146	8.8	1.7		8.6	1.7		8.3	2.2		7.3	2.7	
Total number of children in household	1	91	8.8	1.7	0.9	8.6	1.7	0.9	8.2	2.3	0.8	7.4	2.7	0.8
	> 1	211	8.7	1.7		8.6	1.7		8.1	2.2		7.4	2.6	
Socio-economic status	Lower	146	8.5	1.8	0.01*	8.3	1.8	0.01*	7.6	2.5	< 0.001*	6.8	2.8	< 0.001*
	Upper	156	9.0	1.5		8.8	1.5		8.6	1.9		8.0	2.4	
Past history of injury in the child	Yes	46	9.0	1.3	0.3	8.9	1.4	0.1	8.7	1.7	0.04*	8.0	2.1	0.04*
	No	256	8.7	1.7		8.5	1.8		8.1	2.3		7.3	2.7	

*Significant *p* value (< 0.05)

**Table 5:** Multivariable regression analysis of factors associated with poor perception of hazard, knowledge of type of injury, and perception and knowledge of the prevention of unintentional childhood injuries

Variable	Perception of hazard			Knowledge of type of injury			Perception of prevention			Knowledge of prevention		
	Parameter estimate	95% CI	***p***-value	Parameter estimate	95% CI	***p***-value	Parameter estimate	95% CI	***p***-value	Parameter estimate	95% CI	***p***-value
Intercept	7.36			7.005			6.06			5.07		
Relationship (Mother/others)	−0.52	−1.2,0.23	0.17	0.77	−1.3, 0.24	0.17						
Education (Lower/higher)	0.59	0.15, 1.0	0.008*	0.18	0.33, 1.21	0.001*	0.96	0.4,1.5	0.001*	1.1	0.5,1.8	0.001*
SES (Lower/higher)	0.24	−0.16,0.66	0.23	−0.53	−0.23, 2.4	0.38	0.62	0.08, 1.1	0.02*	0.72	0.08,1.3	0.02*
Past injury (Yes/No)							−0.59	−1.2, 0.91	0.08	−0.74	− 1.5, 0.73	0.07
Model R^2^	0.062			0.082			0.094			0.092		

*Significant *p* value (< 0.05)

### Knowledge of injury and its prevention

Among the participants who had adequate perception of injuries, a vast majority (> 98%) had adequate knowledge on the type of injuries in 8 of the 10 hazardous situations. A lower proportion (< 10%) of them had inadequate knowledge on scenarios such as the child playing with a plastic bag (9.7%) and the child as a pillion on the bicycle (5.1%). While 90% of them were able to suggest preventive measures for 7 of the 10 hazardous situations, a significant proportion of them had inadequate knowledge on prevention for the rest of the scenarios (3/10) such as the child as a pillion rider in a bicycle (28.4%), playing near a construction site (14.1%) and playing with a plastic bag (11.7%). When asked about preventive measures that could be potentially considered in preventing injuries, the most common response was adult supervision in six of the ten scenarios. Some of the preventive measures put forth were:

*“The child must put his/her legs on both sides of the bicycle when riding as a pillion”*

*“Children should not play with firecrackers, and must use personal protective equipment while bursting crackers”*

*“Firecrackers must be segregated according to age for use”*

*“Keep the small children away from the kitchen/cooking area”*

Education of the primary caregiver (*p* = 0.001) and their socioeconomic status (*p* = 0.02) were independently and significantly associated with knowledge on preventive measures for unintentional childhood injuries.

## Discussion

In this study in a rural setting, we interviewed primary caregivers to study their knowledge as well the depth of their perception of common, yet, risky scenarios of unintentional childhood injuries encountered in the community using a photo-elicitation technique.

### Primary caregivers’ perceptions on unintentional childhood injuries

Primary caregivers in this setting had adequate perception of the risk of falls, road traffic injuries, and burns, due to hazardous activities, such as unsupervised climbing of trees, crossing roads in non-zebra crossing zones, and playing with firecrackers, respectively. The perception was comparatively low for situations such as drowning in a bucket of water, suffocation due to plastic bags and burns due to unsafe cooking environment. These observations are similar to studies from India and other LMICs with the main risks identified being stoves being placed within the reach of the child (55.5%) and easy access to the child for open buckets (47.7%) (Khan et al. 2013; Banerjee et al. 2016). A study from Gujarat, India, reported that fire accidents in children frequently occurred when firecrackers were kept within the reach of children. Further, more than two-thirds of the children from low socioeconomic urban settings were at risk of poisoning due to household chemicals and this corroborates with the findings from our study (Chaturvedi 2008). One in ten of our primary caregivers failed to perceive an ungated staircase as a hazard for a toddler, similar to observations made in eastern India where an easily accessible rooftop without a protective barrier was present in a significant proportion of the households, indicating that awareness of the danger posed by the ungated staircase was certainly low in these communities. A similar observation was also made in low socioeconomic urban neighbourhoods of Pakistan where more than half the houses had ungated staircases (Khan et al. 2013; Banerjee et al. 2016). Evidence from the UK suggests that absence of staircase gates increased the risk of falls by 2.5 times in younger children (Kendrick et al. 2016).

### Primary caregivers’ knowledge on injury prevention

Efforts toward injury prevention needs to be multi-pronged, with simultaneous influence made felt along several axes such as community support, legislative initiatives, investments in better infrastructure and personal safety behaviour. Even though safety education could potentially result in safe behaviour, it has been proven that education alone is insufficient to reduce the burden of unintentional childhood injuries, but was effective when combined with legislative initiatives in road-related injuries (Peden et al. 2008; Duperrex et al. 2002). A systematic review of injury prevention interventions for children and young adults, by Towner et al., emphasized the need for synergism resulting from “a variety of approaches including education and training, accessible protective devices and safety equipment, environmental change and legislation along with its enforcement” to reduce the burden of injuries (Towner et al. 2001). Behaviour change theories such as health beliefs, that integrate behavioural analysis with the aforementioned health promotion approaches, incorporating individual, social and environmental factors that influence injury risk, could yield promising results in injury reduction (Gielen and Sleet 2003). Parental/adult supervision of children, along with the implementation of simple safety practices, emerged as the most common preventive measure put forth by the primary caregivers in our study. Though there were multiple risk factors which could contribute to injuries among children, especially under-5 children, lack of supervision is the major contributing factor (Saluja et al. 2004). In general, it is the environmental and behavioural factors that shape the parental attributes which eventually affect the quality of child supervision and the occurrence of injuries (Morrongiello and Kiriakou 2004; Wills et al. 1997). However, evidence also suggests that children are injured even when under close supervision (Schnitzer et al. 2015). The mere presence of primary caregivers at all times may not necessarily imply sufficient supervision of children amidst their household chores, farming, cattle rearing and other activities as highlighted by qualitative studies from LMICs (Siu et al. 2019; Mashreky et al. 2009). This suggests that the population in rural settings, such as ours, require education on simple safety practices to be incorporated along with child supervision.

Our community did not have adequate knowledge of the prevention of injuries on scenarios that involved cooking with stove kept at the ground level, unsafe placement of household chemicals, not following safety rules while crossing the road, and unsafe pillion riding. In India, until 2016, there existed no strict guidelines for mandatory protective headgear for children less than 12 years. However, the recently amended ‘Motor Vehicles Act’ makes it mandatory for even children above 4 years to wear a helmet while travelling on two-wheelers (Deepika 2016; Dash 2016). However, a survey from ten cities in India revealed that 74% of the pillion riders continue to not to wear helmets (Citizen Matters 2019). Thus, India still has a long way to go for the strict enforcement of the same, and a large part of this change will ensue only following education of the population.

We could attribute the adequate perception and knowledge about certain injuries in the Kaniyambadi block as a result of the work done by Department of Community Health, Christian Medical College Vellore, through its Community Health and Development (CHAD) program over six decades. Though CHAD or the government do not have exclusive injury prevention programs, the program has strong community presence through its grassroot workers. Health education sessions as a part of its integrated preventive and curative health programs, have resulted in increase in health literacy and improvement in various health indicators overall in the past few years. Monthly infant mortality reviews and special focus on injury related deaths have also indirectly resulted in an increase in awareness among our study population.

### Specific factors influencing unintentional childhood injuries

Higher educational status of the primary caregivers was associated with a better perception of unintentional injuries in our setting. One in ten of our primary caregivers felt that injuries while riding a bicycle without safety features and burn injuries due to firecrackers cannot be prevented in these settings. A qualitative study from Bangladesh reported that children under-5 years and young adolescent girls involved in cooking were vulnerable to burn injuries, and it was identified that lack of supervision and hazardous environmental settings were the risk factors for these injuries. Primary caregivers in this study felt that poverty and illiteracy was perhaps a hindrance to their practice of safety measures (Mashreky et al. 2009).

Research from the USA and New Zealand have shown that people from low socioeconomic strata were least likely to believe that injuries are preventable (Hooper et al. 2003; Girasek 2001). It was observed in Turkey that those from the lower socioeconomic strata tend to adhere to safety rules lesser than their counterparts (İnce et al. 2017). Studies from South Korea and Taiwan concluded that the deaths due to unintentional injuries in children were associated with living in rural areas and lower parental education (Chou and Chen 2019; Hong et al. 2010). There are few possible explanations that can be given for these oft-reported findings. First, people who have higher income are more educated and have more access to information and hence a better knowledge. Further, this knowledge acquired often reflect the safer environments they live in when compared to those with less resources. Second, evidence points towards the fact that uninsured patients with trauma have higher mortality due to the quality of the care they receive after the injury. Similarly, people with low resources do experience higher rates of fatal injury (Girasek 2001). Financial constraints in low socioeconomic households may preclude them from paying the needed attention to safety precautions, especially if it involves investing money and buying safety devices. In addition to this, other competing health problems in these households also prevent them from perceiving injury prevention as a necessary priority (Girasek 2001).

### Context-driven safety measures: a need of the hour

It’s known that communicable diseases are highly prevalent among children in low- income settings and hence, investment in injury prevention initiatives in the household tend to take a back seat as they are considered as “accidents” and “acts of God”. However, in spite of these constraints a few inexpensive, context-driven safety precautions could be practiced in these settings. For example, injuries to a child playing with household chemicals could be prevented by storing the chemicals beyond the child’s reach. It has been proven that use of helmets significantly reduces mortality in road injuries in LMICs and HICs (Toroyan and Peden 2007). While safety education alone is not sufficient to reduce the road traffic injuries but when combined with legislations such as child restraints and drinking laws it has been found to be highly effective (Peden et al. 2008; Duperrex et al. 2002). A child encountering injuries at a non-zebra crossing could be prevented by teaching the child about safe road practices by incorporating innovative methods such as story-telling with storybooks, using bilingual pictorial books, and role-playing as evidenced by a study among school children in Pakistan (Ahmad et al. 2018).

The major sources of burns among children in these geographical location and in other LMICs are are hot liquids, lamps and stoves. Floor level cooking is a common practice in rural India as these households are constrained with space and money to construct cooking platforms. Hence, floor level cooking is usually done outside the house which eventually increases the incidence of burn injuries. This can be prevented by financial support by the government to make cooking platforms or making safe cooking areas with barricades. Similarly, hanging handle-holders, safe lighting sources and stoves may also prevent burn injuries. Drowning can be prevented by emptying the vessels and containers with water or covering them, fencing or using barriers to the water storage areas and ponds (Alonge and Hyder 2014). Basic wooden or bamboo barricades could make staircases inaccessible and prevent fall from heights.

### Future pathway

It is also the need of the hour to develop context-specific programs for the community such as the “Supervising for Home safety” program developed by Morrongiello which showed a positive impact through parent injury risk appraisals, as compared to a control group, and these changes persisted through 1 year following the intervention (Morrongiello et al. 2013). Morrongiello also noticed changes in other aspects of parental supervision, such as a decrease in the unsupervised time of their child and an increase in the level of supervision, with these changes persisting until 3 months after the intervention (Morrongiello et al. 2013). This also calls for periodic reinforcement in the community, which when delivered through initiatives amalgamated with the existing maternal and child health programmes would be impactful. Another successful intervention from Bangladesh is worth noting: a package of community-led crèche interventions for drowning prevention was associated with 29 and 28% reduction in hospitalization and mortality, respectively, among children aged 0 to 17 years. Similarly, these interventions also brought down deaths due to drowning by 44% from the baseline rate among the children aged 9 to 47 months (Alonge et al. 2020; Evaluation of PRECISE: a comprehensive child injury prevention program in Bangladesh 2006-2008).

### Strengths and limitations

This is the first community-based study which used a photo-elicitation method to quantify perception related to unintentional injuries. The major strengths were the robust sample size and a novel approach used in a rural setting. Since this was a quantitative study, and the sample size was large given the limited time frame, the investigation was not at a sufficient depth to get a complete perspective on each hazardous scenario, and in-depth interviews could not be performed with the participants. As the interviewers and the participants were new to the photo-elicitation method, data collection was time consuming. A standardised validated scoring system could not be used for this study to determine right responses or to categorize the open-ended responses, due to a lack of similar studies that employed a similar methodology. We were also unable to validate our study tool before the data collection. Though our study brings out significant findings from a rural settings in India, it cannot be extrapolated to the entire country as Kaniyambadi block from Vellore region where the study was conducted is an exemplar of outstanding public health outcomes because of the efforts and work done by Christian Medical College, Vellore over hundred years.

Probably, in-depth interviews with a smaller sample size using a similar photo-elicitation method with a qualitative approach may improve our understanding about caregivers’ perceptions of unintentional childhood injuries. Further, the establishment of national unintentional childhood injury prevention surveillance networks could shed more light towards the better understanding of the same in wide-varied settings.

## Conclusion

Primary caregivers in this rural setting generally have an adequate perception of unintentional childhood injuries. However, in areas related to road traffic injuries, suffocation and burn injuries they revealed inadequate knowledge, indicating these as potential areas for improvement. Better education and socioeconomic status were associated with better perception and knowledge of unintentional childhood injuries and their prevention. To prevent unintentional childhood injuries, we recommend the incorporation of community-based interventions into the existing maternal and child health programs, focusing on road traffic injuries, burns, and suffocation. These interventions must aim at behavioral change at the level of the primary caregivers by adopting simple safety practices in the home environment, along with a holistic yet carefully tailored approach for the overall community.

## Data Availability

Datasets are available with the corresponding author at request.
